# Pilar Cyst From a Maxillofacial Surgeon’s Perspective: A Case Report and Review of Literature

**DOI:** 10.7759/cureus.38508

**Published:** 2023-05-03

**Authors:** Srinidhi Kasthurirengan, Senthil Murugan Pandurangan, Mahathi Neralla, Murugesan Krishnan, Bala J Gupta

**Affiliations:** 1 Oral and Maxillofacial Surgery, Saveetha Dental College, Chennai, IND; 2 Oncology, Oral Cancer Institute, Saveetha Dental College, Chennai, IND

**Keywords:** epidermoid cyst, trichilemmal cysts, temporal region, osteomyelitis, pilar cyst

## Abstract

Pilar cysts/trichilemmal cysts are benign lesions that arise from the hair follicle epithelium. The commonest area of occurrence is the scalp but it can also occur in the head and neck region. The pilar cyst grows at a prolonged rate. They are relatively rare and occur in about 10% of the population. They appear in the region of high concentrations of hair follicles. A 75-year-old male patient came to the Department of Oral and Maxillofacial Surgery with a chief complaint of swelling on the left side of the face for the past one year. Cytological examination revealed an infected cystic lesion. Computed tomography (CT) showed a well-defined lesion in the left temporal region. After surgical excision of the lesion, it was sent for histopathological examination. Excisional biopsy revealed a pilar cyst. We report a rare case of pilar cyst in the left temporal region in a patient who was previously operated on for osteomyelitis of the left side of the mandible up to the coronoid process. These cysts may mimic temporal space infection and lead to an incorrect treatment plan.

## Introduction

Pilar cysts, otherwise known as trichilemmal cysts, are benign growths that arise from the hair follicle epithelium. They are most commonly seen on the skin of the scalp but can also occur in other areas of the head and neck regions [1]. Pilar cysts occur in about 10% of the population. They are usually non-syndromic and sporadic. These are the most common types of skin cysts [2]. These cysts are more common in areas with a high concentration of hair follicles. These are the most common cutaneous cyst in the scalp and the second most common cysts in the head and neck. Pilar cysts are benign in nature and rarely transform into malignant lesions. In about 2% of pilar cysts, single or multiple proliferating cells lead to malignant tumours, often called proliferating trichilemmal cysts or tumours [3].

Trichilemmal cysts could be passed down via the autosomal dominant gene. Patients with familial pilar cysts are typically younger and have multiple lesions at the same time. They develop from the epithelium between the sebaceous gland and the arrector pili muscle. They are most common on the head, especially the scalp. The pilar cyst grows at a very slow rate; it takes several years to grow to a large size [2]. 

In this case report, we present a rare case of a pilar cyst in the left temporal region of a 75-year-old male with a history of surgery for osteomyelitis of the left mandible extending up to the coronoid process. This cyst was mimicking temporal space infection in the left temporal region.

## Case presentation

A 75-year-old male presented to the Department of Oral and Maxillofacial Surgery with a history of swelling in the left temporal region that had been slowly growing for the past one year. The patient complained of mild pain and discomfort at the site of the swelling. The patient also complained of restricted mouth opening. The patient also had a history of chronic suppurative osteomyelitis of the left mandible, which was operated on a year ago. The patient had no significant medical history and was not on any medications.

**Figure 1:**
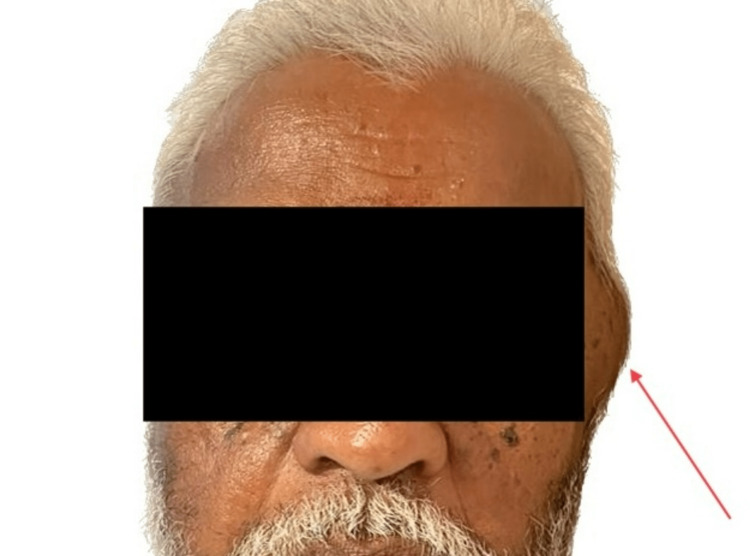
Preoperative image of the swelling. The image shows swelling in the left temporal region preoperatively.

**Figure 2:**
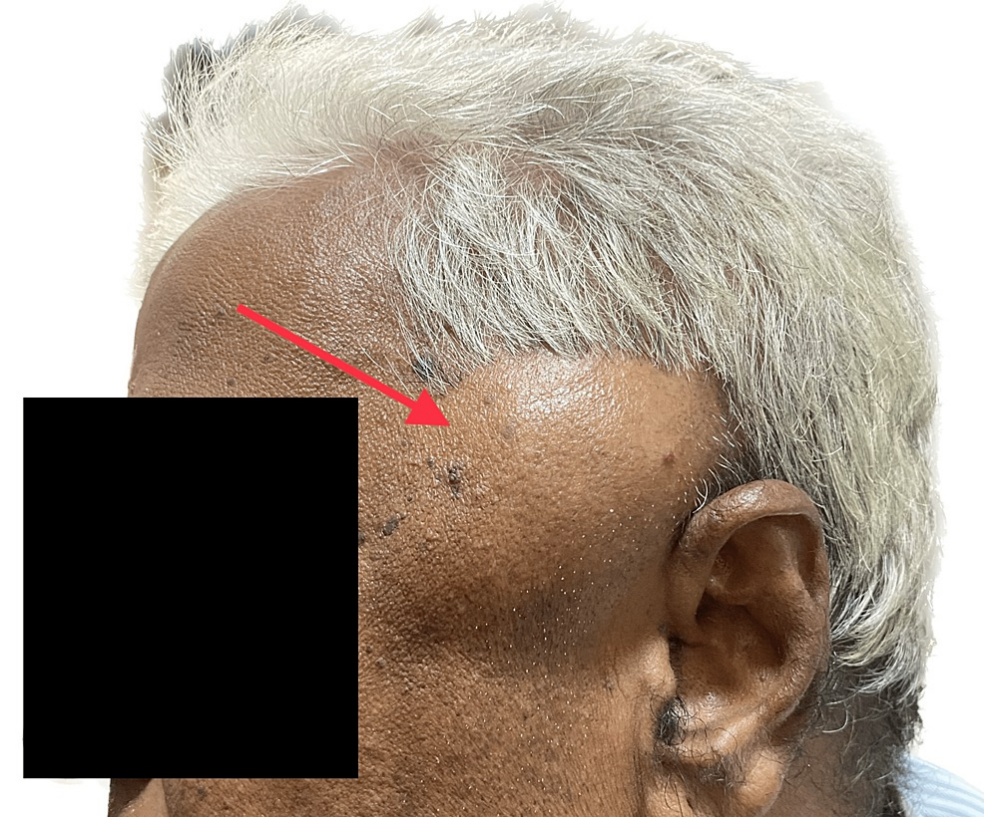
Preoperative profile view of the swelling. The image shows a profile view of the lesion on the left temporal region. Note the skin over the swelling appears normal.

On examination, a soft, non-tender, fluctuant swelling measuring 4.5 × 3.0 cm was noted in the left temporal region (Figures 1, 2). The overlying skin was normal and appeared intact, without any signs of inflammation. No neurological or sensory/motor deficits were noted. Restriction of mouth opening was noted. All these clinical features were mimicking temporal space infection except for the duration of the swelling. 

**Figure 3:**
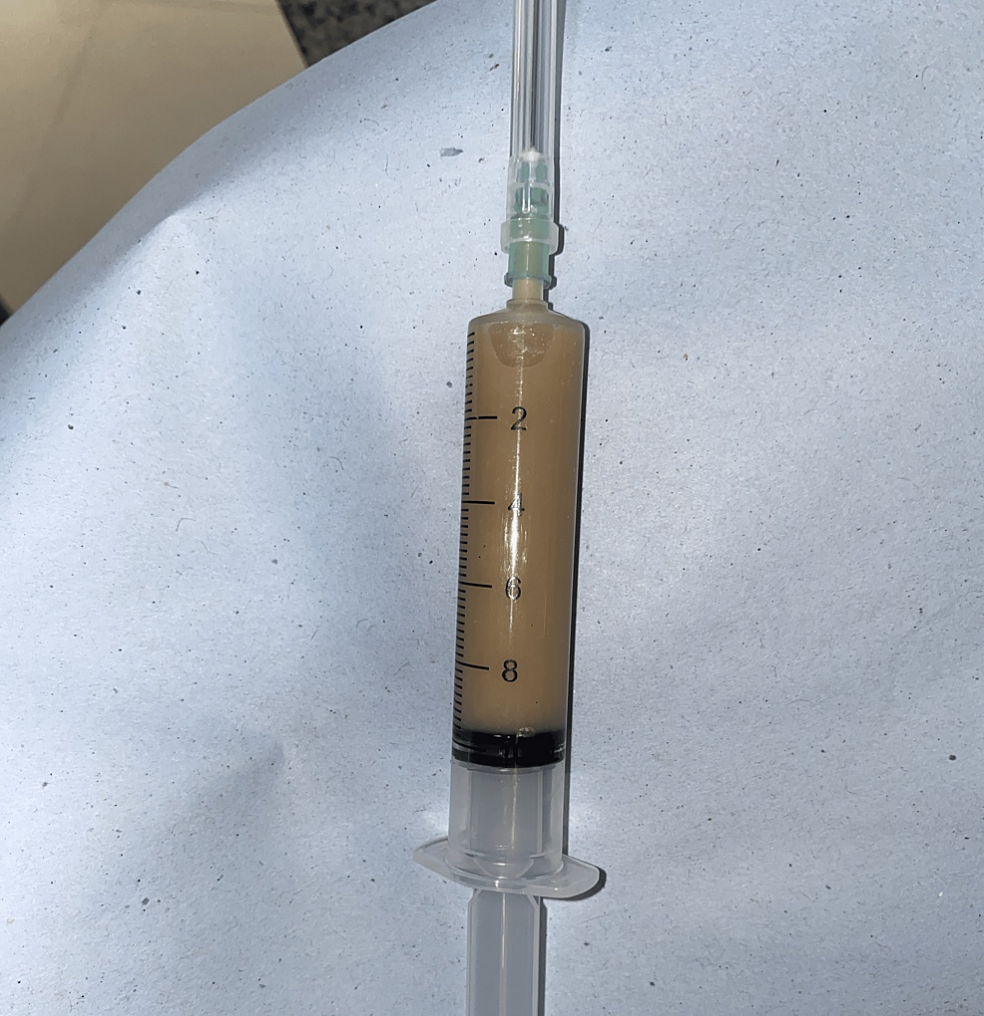
Needle containing aspirate from the lesion. The image shows dirty brown pus-like material that was yielded from the lesion.

**Figure 4:**
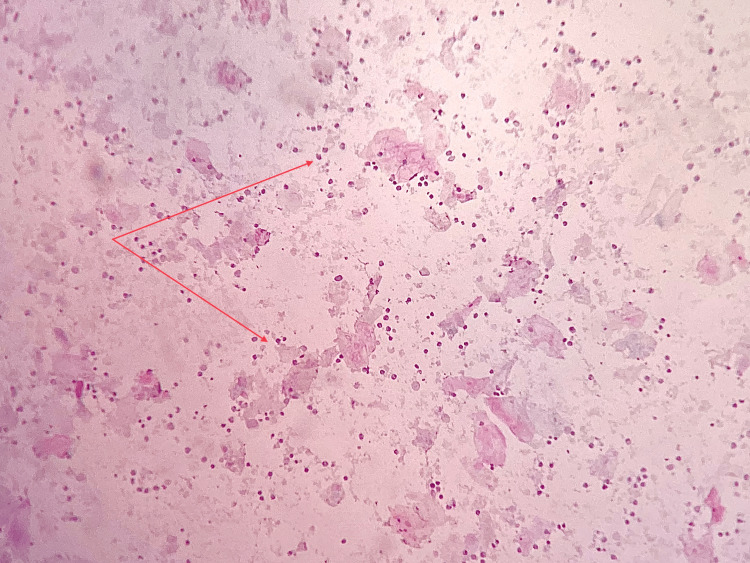
Fine needle aspiration cytology showing nucleated and anucleated cells along with mixed inflammatory cells (arrow) (MGG, 10x)

**Figure 5:**
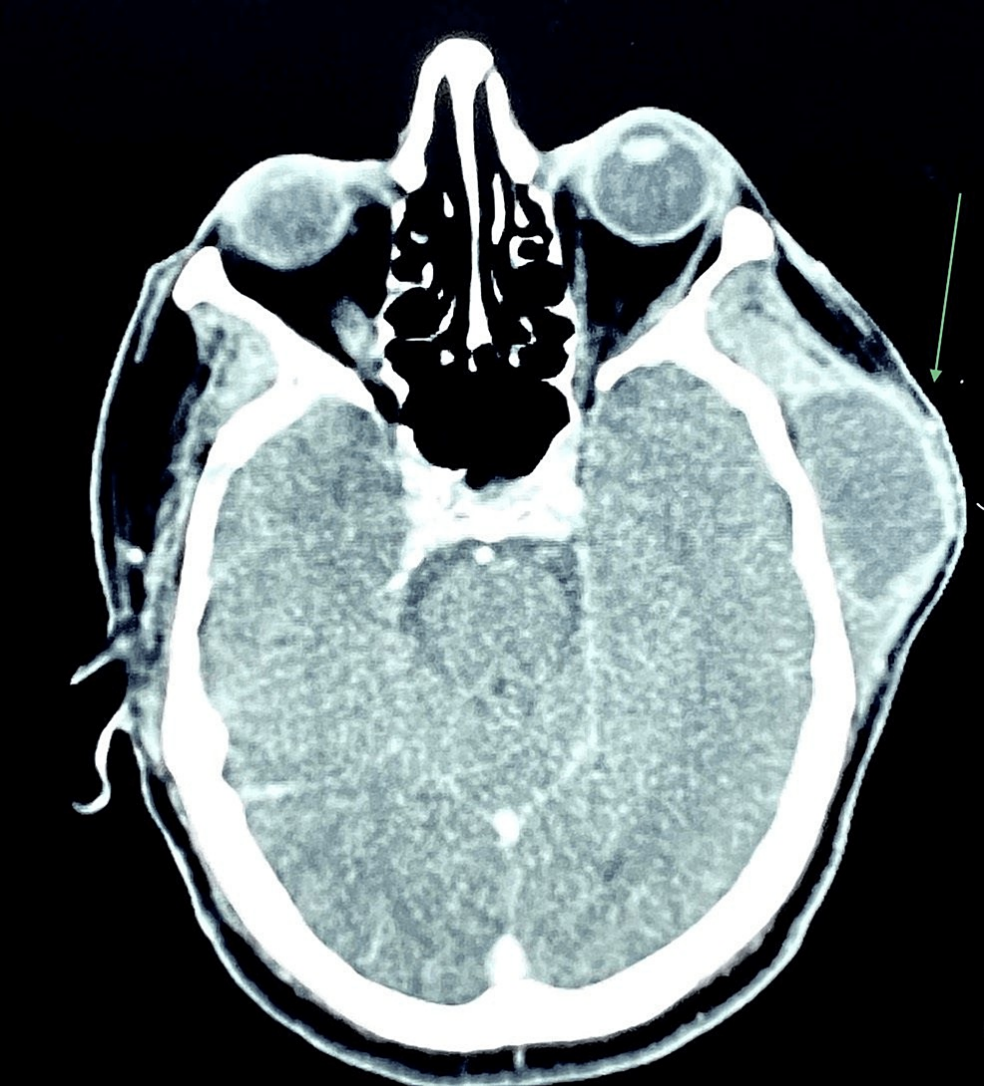
Section of Computed Tomography (CT) showing lesion in the left temporal region. The arrow in the axial view of the CT shows a well-defined cystic lesion in the left temporal region.

**Figure 6:**
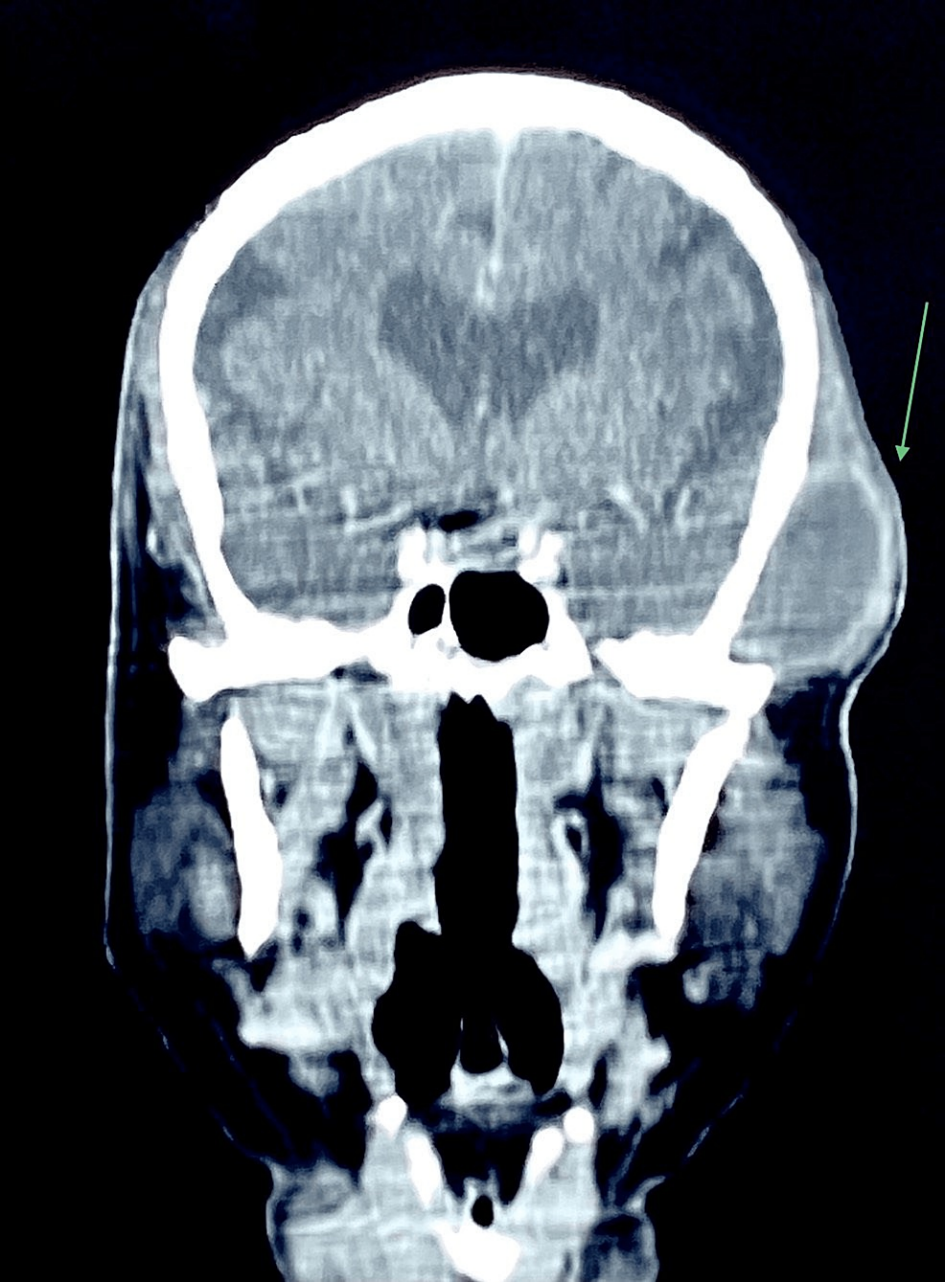
Section of Computed Tomography (CT) showing lesion in the left temporal region. The arrow in the coronal view of the CT shows a well-defined cystic lesion in the left temporal region.

Fine needle aspiration cytology was performed, which yielded dirty brown pus-like material (Figure 3). Cytology results revealed it as an infected cystic lesion. No malignant cells were identified on cytological examination. Abundant nucleated and anucleated squamous cells admixed with mixed inflammatory infiltrate predominantly neutrophils and lymphocytes (Figure 4). A computed tomography (CT) scan of the head and neck showed a well-defined, cystic mass in the left temporal region just beneath the temporalis fascia (Figures 5, 6). Routine investigations, including complete blood count, serology, liver function tests, and Chest X-ray were within normal limits. The patient was evaluated by the anesthesiologist and found to be fit for surgery. 

**Figure 7:**
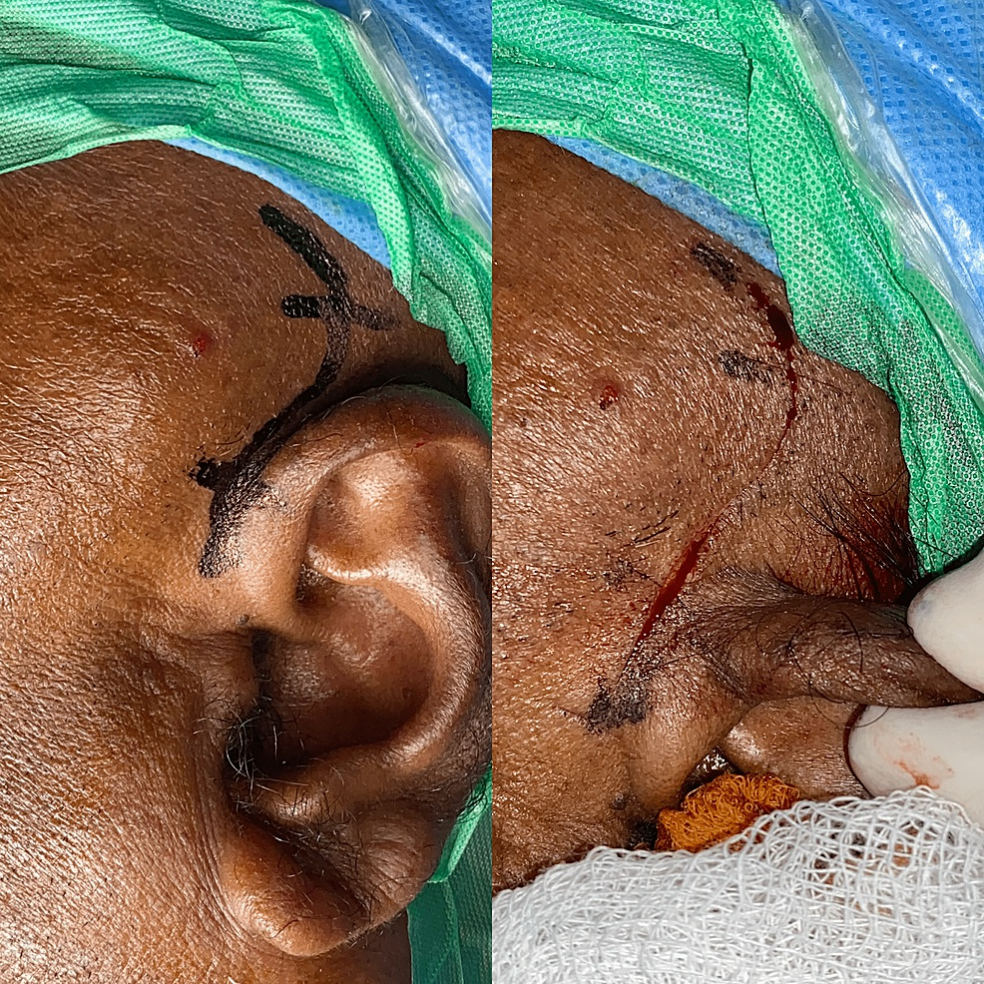
Popowich incision marking and incision placement The image on the left shows the marking of the Popowich incision and the image on the right shows the placement of incision.

**Figure 8:**
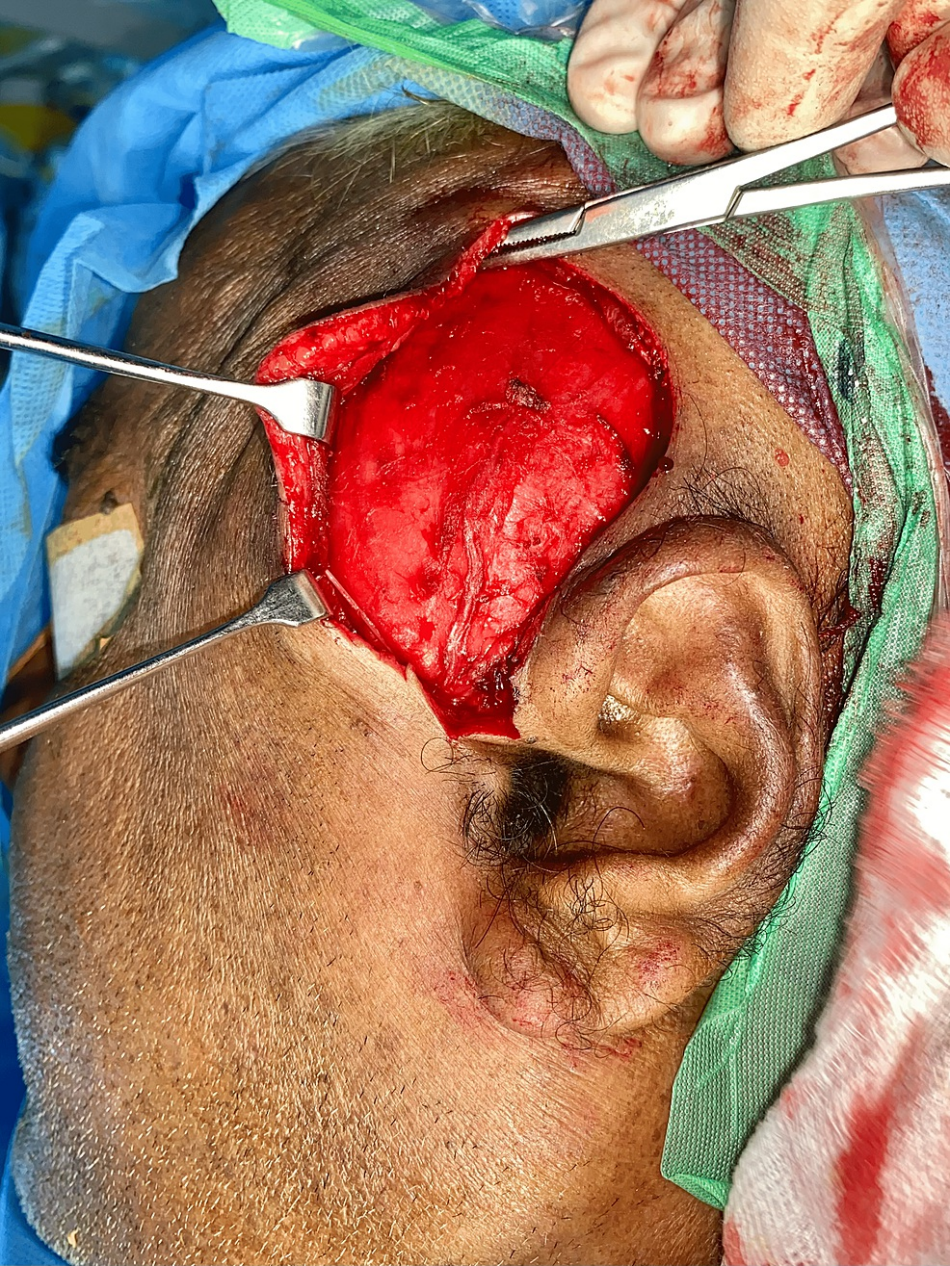
Exposure of the lesion The image shows the dissected and isolated lesion in the left temporal region

**Figure 9:**
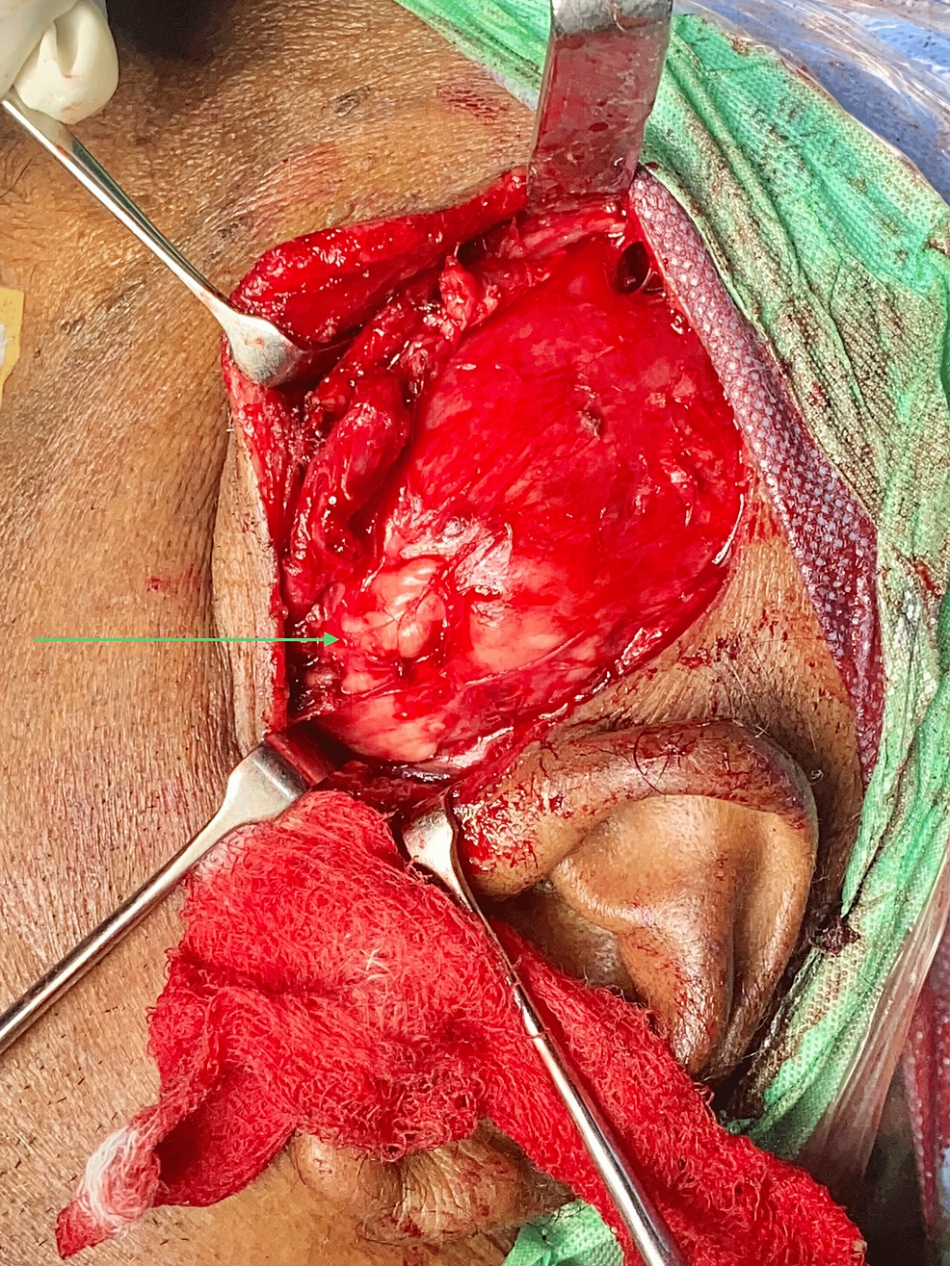
Lobulated surface of the lesion. The arrow in the image shows a whitish lobulated surface.

**Figure 10:**
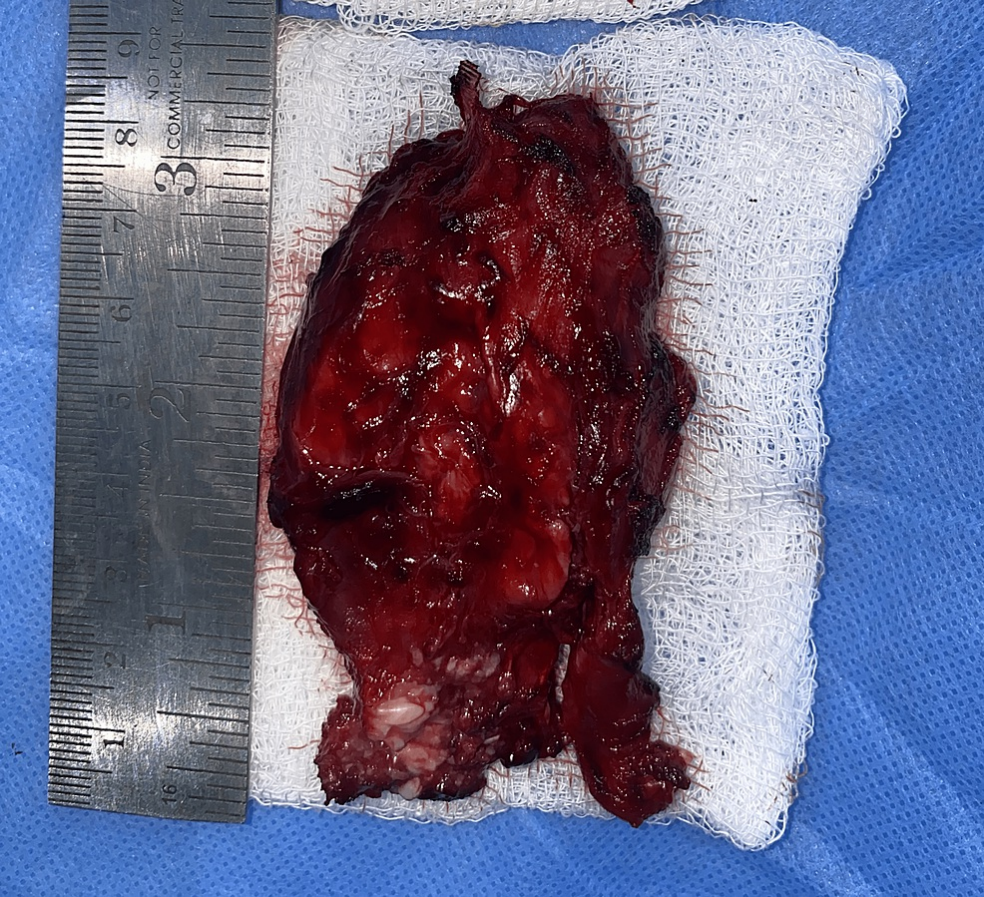
Excised specimen

**Figure 11:**
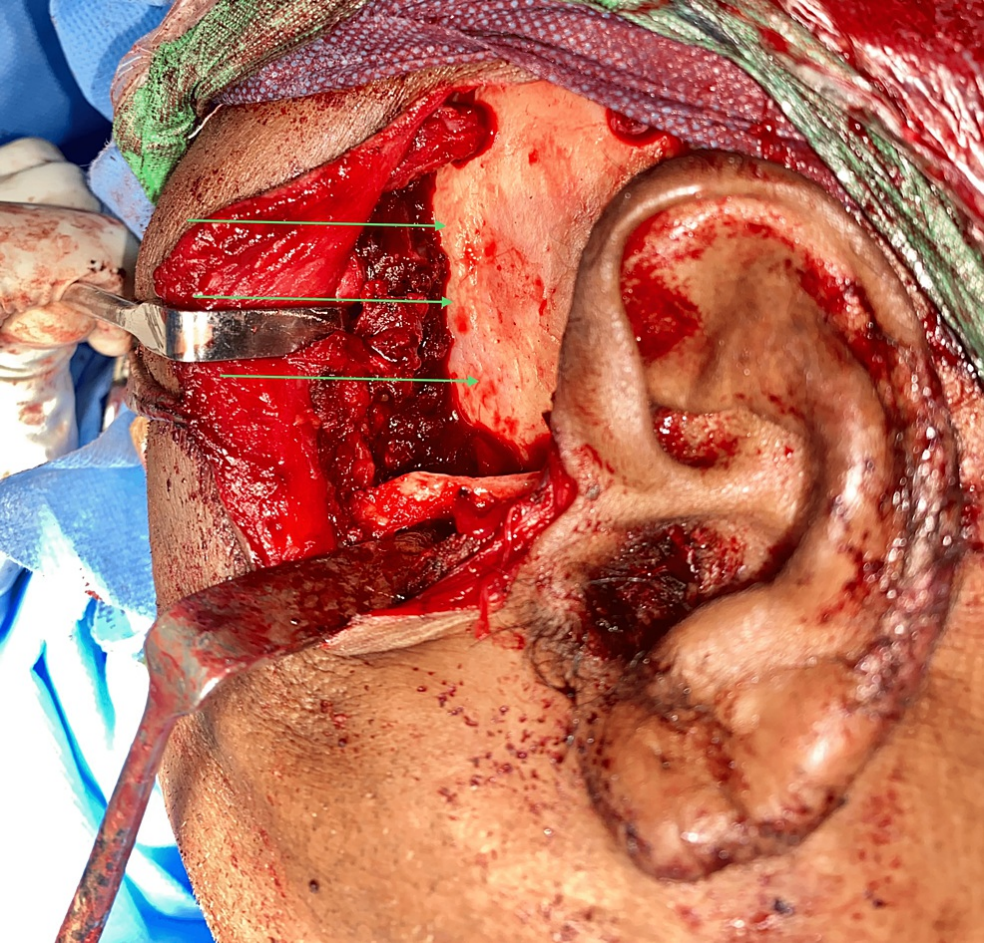
Crater-like defect in the left temporal region. The arrows in the image show crater-like defects due to pressure erosion caused by the lesion.

**Figure 12:**
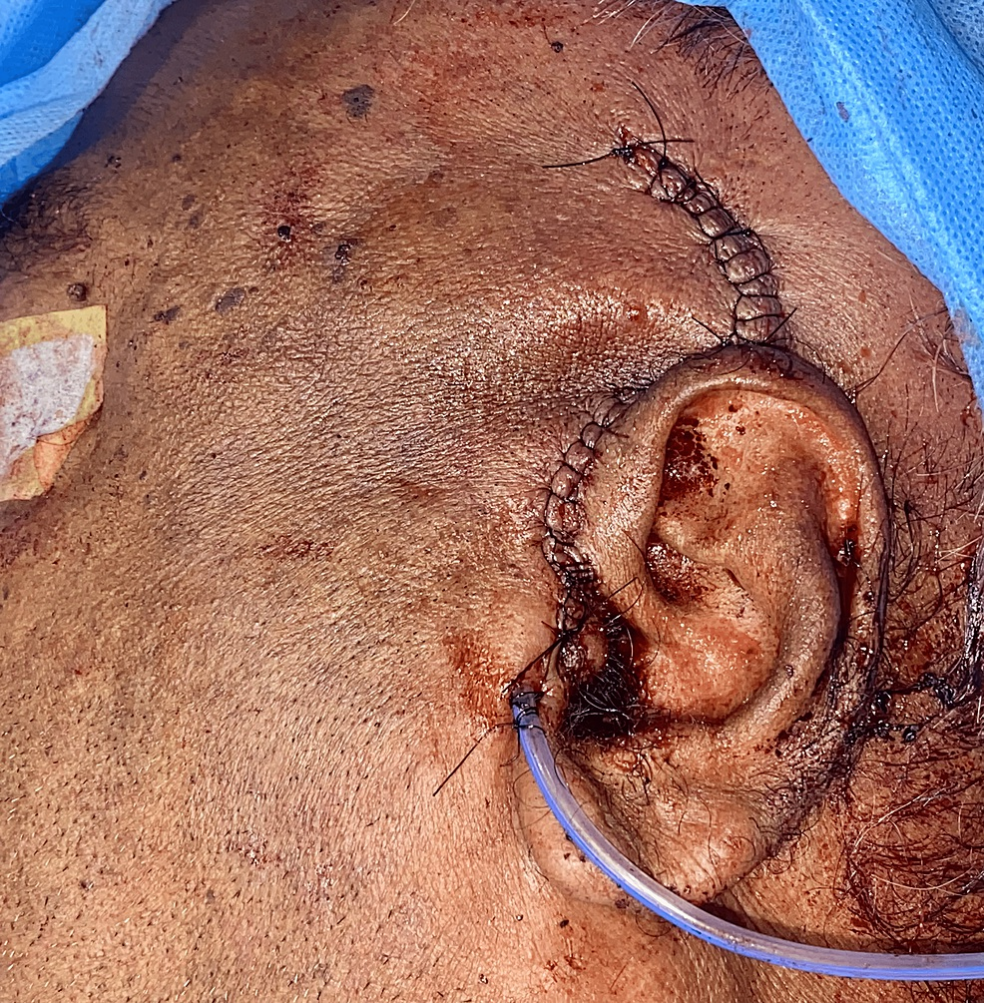
Closure of the lesion. The image shows closure of the lesion and placement of the drain.

The patient was counselled about the benign nature of the lesion and the possibility of surgical excision. Informed consent was obtained from the patient, and the surgery was scheduled. Under general anaesthesia, nasoendotracheal intubation was done, and standard scrubbing and draping were done. Two percent Lignocaine with adrenaline 1:100000 was given as local infiltration. Popowich incision given (Figure 7), layer dissection done through temporalis fascia (superficial and deep fascia), cystic lining identified near the zygomatic arch and dissected along with atrophied temporalis muscle and fascia (Figure 8), lesion measuring 7.6 x 4.8 x1.6 cm was isolated. The lesion had a lobulated surface (Figure 9). It was removed in toto (Figure 10). There was a notable crater-like defect on the temporal bone due to the compression caused by the lesion (Figure 11). This may denote the long-standing nature of the lesion. It was noted that the lesion was extending below the zygomatic arch and the extension was also included in the specimen. The closure was done in layers using 3-0 vicryl (Polyglactin) and 4-0 ethilon (Nylon) in a simple interrupted technique (Figure 12) [4]. A mini vac drain was placed. Hemostasis was achieved. Extubation was uneventful. The patient was reviewed in the post-operative period, with no signs of facial nerve weakness and paralysis. The patient had an uneventful postoperative course and was discharged two days after the surgery.

**Figure 13:**
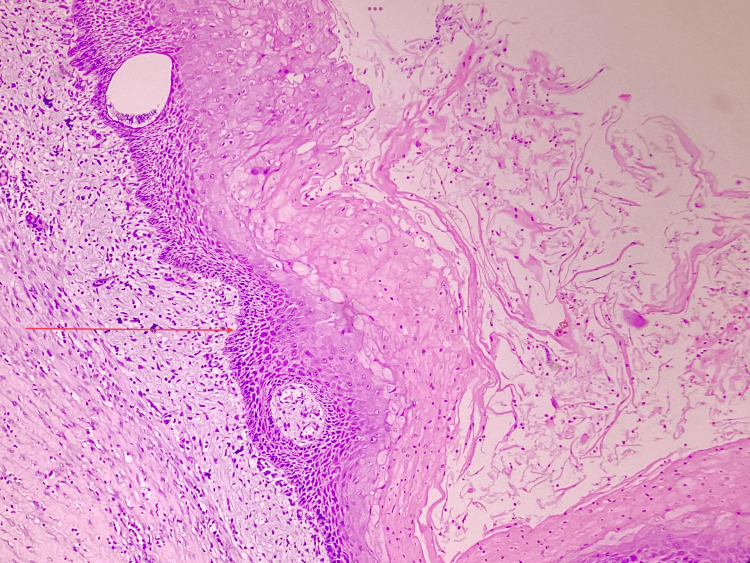
Histopathology of the cyst wall shows stratified squamous epithelium with absent granular layer and abrupt compart keratin filling the cyst lumen (H & E, 20x) The arrow in the images shows the variations of the epithelium.

The excised specimen given for histopathological examination revealed multiple sections of tissue which appear cystic in architecture and are lined by hyperparakeratinized stratified squamous epithelium with areas of confluent rete pegs formation. In some areas, the epithelial lining appears stretched. The basal cells are cuboidal to low columnar. The superficial spinous layer abruptly transitions to a dense compact layer of parakeratin filling the lumen without intervening granular cell layer. The supporting dense connective tissue wall shows sub-epithelial dense inflammatory infiltrate along with few satellite cysts and foreign body type giant cell reaction. There is also the presence of moderate vascularity, nerve bundle, skeletal muscle, and adipose tissue along with areas of haemorrhage. At sites, focal inflammatory infiltrate is seen extending into the skeletal muscle. The central portion of the cyst contained keratinous material, consistent with the diagnosis of a pilar cyst (Figure 13). The histopathology lacked microorganisms, congested or thrombosed blood vessels, and infiltrates of neutrophils, which are the consistent finding in osteomyelitis and helps to confirm the diagnosis.

On follow-up after three months, the patient had no recurrence of the swelling or any major complications. The patient was advised to regularly follow up to monitor the area for any signs of recurrence.

## Discussion

A pilar cyst, also known as an isthmus-catagen cyst, develops from the follicular isthmus' exterior root sheath. Around 20% of epithelial cysts are pilar cysts; the remainder are epidermal cysts [5]. Pilar cysts are relatively common benign lesions that most frequently arise on the scalp [1.3]. These cysts are thought to arise from the infundibular portion of the hair follicle epithelium. Pilar cysts are usually asymptomatic and slow-growing. These cysts can occur in any age group, but the incidence increases with age. The occurrence of pilar cysts in the temporal region is rare, and the presence of a pilar cyst in a patient with a history of osteomyelitis of the mandible is extremely rare [2]. Rare cases of the oral and maxillofacial region [6-15] require accurate clinical assessment, proper diagnosis, and appropriate treatment. 

Our patient presented with a benign, non-tender mass in the left temporal region, which was confirmed via needle aspiration and a CT scan of the head and neck. The patient underwent a successful surgical excision with no postoperative complications. A histopathological examination of the cystic mass confirmed it to be a pilar cyst. At first, this lesion mimicked a temporal space infection, which was misleading. But after performing the fine needle aspiration cytology, it was found to be an infected cystic lesion. Thus the present case emphasizes the important role of fine needle aspiration cytology in any swelling. However, histopathology is necessary to confirm the diagnosis of a pilar cyst especially to rule out its closest differential of epidermal inclusion cyst [16].

Surgical excision is considered the standard treatment for pilar cysts. The procedure is cosmetic and can be done under local anaesthesia or general anaesthesia. In our case, the surgery was done under general anaesthesia. Tissue dissection should be done carefully to prevent spillage of the cystic contents, which could lead to an acute inflammatory response, a foreign body reaction or recurrence. The most important point to be noted here is the crater-like defect that was present on the temporal bone after the excision of the lesion. This defect shows that the lesion was present for a long time.

Our patient had a rare coincidence of a pilar cyst in the left temporal region, which is an unusual site of occurrence, along with a history of osteomyelitis of the mandible. Although the presence of a pilar cyst is not related to osteomyelitis, taking a thorough medical history and performing regular follow-ups in such cases is essential for prompt diagnosis and management.

## Conclusions

Pilar cysts are benign lesions that may occur in various regions of the head and neck area. Surgical excision remains the standard treatment for these cysts, with minimal risk of recurrence. In this article, we would like to conclude that the occurrence of a pilar cyst in a patient with a history of osteomyelitis of the mandible is rare, but taking a thorough medical history in such cases is crucial for prompt diagnosis and management. Regular follow-ups are essential for monitoring the area for any signs of recurrence or complications. Even though such cases occur in rare instances, we, as oral and maxillofacial surgeons, need awareness regarding such lesions for proper diagnosis and to provide the appropriate treatment to the patients.
